# The selection and definition of indicators in public health monitoring for the 65+ age group in Germany

**DOI:** 10.25646/5990

**Published:** 2019-06-27

**Authors:** Maike M. Grube, Christa Scheidt-Nave, Beate Gaertner, Denise Lüdtke, Michael Bosnjak, Stephanie Heinrich, Nils Lahmann, Gabriele Meyer, Kilian Rapp, Steffi Riedel-Heller, Martina Schäufele, Karin Wolf-Ostermann, Susanne Zank, Judith Fuchs

**Affiliations:** 1 Robert Koch Institute, Berlin; 2 Leibniz Institute for Psychology Information Trier; 3 Martin Luther University Halle-Wittenberg; 4 Charité-Universitätsmedizin Berlin; 5 Robert Bosch Hospital, Stuttgart; 6 Leipzig University; 7 Mannheim University of Applied Sciences; 8 University of Bremen; 9 University of Cologne

**Keywords:** PUBLIC HEALTH, SURVEILLANCE, AGE, INDICATORS, HEALTH MONITORING

## Abstract

Selecting relevant indicators is an important step in the development of public health monitoring for older people. Indicators can be used to combine information comprehensively from various data sources and enable recurring, comparable findings to be made about the health of older people. Indicators were systematically compiled from existing international monitoring systems. An indicator set on health in old age was developed using a multistage, structured consensus-based process together with an interdisciplinary panel of experts. The resulting 18 indicators were assigned to three health areas: (1) environmental factors, (2) activities and participation, and (3) personal factors. Data sources that can be used for the indicators are the health surveys within the framework of the Robert Koch Institute’s (RKI) health monitoring system, as well as surveys from other research institutes and official statistics. In the future, the indicator set is to be developed further and integrated into an overall approach that is geared towards health reporting and the monitoring of chronic diseases in all phases of life.

## 1. Introduction

Public health monitoring involves the systematic and continual provision of health-related information from various data sources that enables health care stakeholders and health and social policy makers to use the best available evidence to make decisions [1, 2]. In its 2017 Global Strategy and Action Plan on Ageing and Health [3], the World Health Organization (WHO) describes the implementation of a sustainable and efficient system to monitor the health of older people as a priority area for improving health in old age. Moreover, the report stresses that agreement on important core concepts and internationally comparable measures is essential. However, such agreement requires open debate about health priorities and values, and needs to involve key stakeholders, including older people. Despite the fact that promising indicator-based approaches to health monitoring for older people have already been advanced internationally [4, 5], Germany has yet to see comparable developments at the federal level.

The Improving Health Monitoring in Old Age (IMOA) project took place between 2016 and 2018 and was funded by the Robert Bosch Stiftung. The project set itself the goal of developing a concept for a nationwide system of public health monitoring that would cover the entire population aged 65 or above. Moreover, it was aimed at developing a conceptual framework and to select relevant indicators that would permit recurring and comparable health information to be made available for the population aged 65 and above. In addition to using data from the nationwide examination and interview surveys regularly undertaken as part of the health monitoring at the Robert Koch Institute (RKI), data from other sources, such as registry data and health insurance administrative data need to be considered in an indicator-based health information system to a greater extent, as these data are available regularly and cover all age groups [6].

This paper describes the selection and definition of indicators for a public health monitoring of the older population in Germany.

## 2. Methodology

Indicators were selected together with an interdisciplinary panel of experts consisting of fifteen appointees and two alternates from the fields of general practice, geriatrics, gerontology, public health, survey methods and nursing science as well as from a civil society organisation (Annex Table 1). In line with the WHO’s World Report on Ageing and Health [7] and the International Classification of Functioning, Disability and Health (ICF) [8], the following three areas on health in old age were agreed upon: (1) environmental factors, (2) activities and participation, and (3) personal factors. These areas provided the overall framework for further indicator development. In March 2017, a joint one-day workshop – supported by a qualitative content analysis of national and international health goals for older people – chose relevant topics from each of the three areas on which the indicators were to focus [9]:

1. Health care provision, nursing and community care, physical environment, and social environment
2. Social participation and activities of daily living
3. Physical health, mental health, physical and cognitive functioning, and health behaviour

The systematic inventory of existing indicator sets was followed up by a multistage, structured consensus-based process that was used to select and define relevant indicators with which to monitor health in older age.

### 2.1 Indicator research

Between June and July 2017, comprehensive research was conducted into national, indicator-based monitoring systems of health in older age. A detailed description of the study’s methods and findings has been published elsewhere [10]. The research was limited to the 35 member states of the Organisation for Economic Co-operation and Development (OECD). Indicator sets were only taken into account if they were written in English or German, had been published or updated after 1 January 2007, if data were available from more than one source, for example from survey data and from health insurance administrative data, and if information was available about how the indicators were being operationalised. No restrictions were placed on a particular format, meaning that indicator sets were accepted as reports, brochures, web pages or scientific papers. The research focused on the websites of national public health institutes, involved a supplementary search of the Internet using the Google search engine, as well as a literature review (via PubMed) of the Medline electronic database. Ten sets of indicators from Finland, the United Kingdom, Ireland, New Zealand, Switzerland and the US met the previously-defined criteria for inclusion, and their structure, related development processes and content were subsequently analysed.

### 2.2 Indicator selection and evaluation

Two independent reviewers assessed and evaluated the indicators identified by the research. Indicators that fitted into the previously-defined conceptual framework for indicator selection were included in the next step. In addition, the following exclusion criteria were defined: (1) duplicates of content, (2) indicators that were not fully compatible with the German health or social care system, (3) indicators that were not clearly worded and, thus, were difficult to interpret, and (4) indicators that were used for regional comparisons only and, therefore, could not be aggregated to the national level. The remaining indicators were supplemented by indicators proposed by the RKI project team that had not been included in existing monitoring systems but that provided information about the topics that had been given priority in the development of the conceptual framework.

This step was followed by a structured consensus-based process that was carried out as a three-stage modified Delphi technique based on an approach developed by the EU-initiated and funded Joint Action on Chronic Diseases initiative [11].

During the first stage, which took place between October and November 2017, the fifteen members of the expert panel were asked to use a 9-point scale (1 = low relevance; 9 = high relevance) to rate the indicators. On this account, the experts were provided with a standardised evaluation form via e-mail. The panel was able to use this form to include additional notes and to raise questions about the indicators.

The panel used the following criteria to assess the indicators [12]:

▶ Higher indicator values point to improved health-related quality of life and/or a healthy life expectancy among older people.
▶ Higher indicator values point to reduced health inequalities among older people.
▶ Indicators can be influenced by policy measures or public health interventions.
▶ Indicators are meaningful and relevant for the public and for stakeholders from the fields of politics and health care.
▶ The indicator is easy to understand and interpret.
▶ The indicator is valid and reliable – it measures what it is intended to measure.

**Figure 1:**
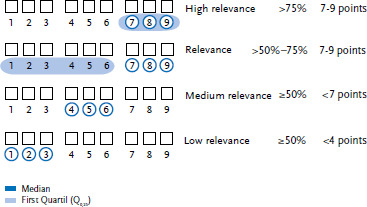
Grading system to rate indicators according to their relevance for public health monitoring for the 65+ age group Own diagram

Fourteen of the fifteen evaluation forms were filled in and returned to the RKI. Each potential indicator was ranked according to the distribution of the points given in the evaluation sheets, taking into account the median and the first quartile (Q_0.25_) (Figure 1):

▶ Indicators were classified as highly relevant if more than 75% of the ratings were in the top range (7-9 points), i.e. the median and the first quartile (Q_0.25_) were 7-9 points.
▶ Indicators were classified as relevant if more than 50% and less than 75% of the ratings were in the top range (7-9 points), i.e. the median was 7-9 points and the first quartile (Q_0.25_) was below 7 points.
▶ Indicators were classified as of medium relevance if at least 50% of the ratings were in the lower (1-3 points) and medium (4-6 points) range, i.e. the median was below 7 points.
▶ Indicators were classified as of low relevance if at least 50% of the ratings were in the lowest range (1-3 points), i.e. the median was below 4 points.

On 15 December 2017, the experts were invited to Berlin to take part in a one-day workshop – the second stage of the Delphi technique. Nine of the fifteen experts participated. The workshop began with a presentation of the results of the first stage. This also included a discussion of the questions and proposals regarding the individual indicators that had been raised on the evaluation forms. At the end of the workshop, the participants were once again asked to provide a written assessment of the indicators that had been classified as either highly relevant or relevant during the first stage. The evaluation was carried out in writing and the format of the evaluation sheets was identical to that used in the first stage.

Indicators that had been classified as highly relevant during the second stage of evaluation (those where at least 75% of the ratings were in the top range – between 7 and 9 points) went on to the third and final stage of the Delphi technique. Indicators that the participants had not classified as highly relevant were excluded in order to concentrate on those that they considered as most important. However, after reconsidering the scientific evidence, the participants requested a reassessment of some of the indicators that had been categorised as of medium relevance during the first stage. In line with the framework provided by the European Core Health Indicators (ECHI) [13], the following factors were taken into account while preparing the indicator set: definition, available data sources, type and periodicity of the data sources, reference population, and the possibility of stratification by gender and socioeconomic status. In addition, a short summary was drawn up of each indicator’s scientific background, which also included a list of important references. The resulting set was presented to the experts who were then asked to re-evaluate the indicators and to provide their views in writing (per e-mail). The experts used a 9-point scale and were able to make proposals, provide criticism and call for changes to be made to the proposed operationalization and listed data sources. Eleven of the fifteen evaluation sheets were returned to the RKI as part of the third evaluation stage.

## 3. Results

**Figure 2:**
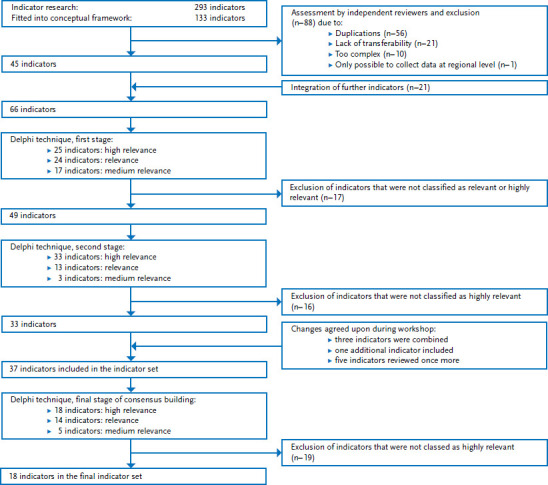
Indicator selection for public health monitoring of the 65+ age group Own diagram

The final indicator set contains 18 indicators (Table 1). Figure 2 summarises the selection process. The results of the structured consensus-based process on which the selection of indicators is based are set out in detail in the Appendix (Annex Table 2).

As part of the indicator research, ten indicator systems with a total of 293 individual indicators were identified using the described search strategy. A total of 133 of these indicators were assigned to one of the previously-defined topics. Of these, 56 indicators were excluded as duplicates, 21 indicators because they were not fully compatible with the German health or social care system, and ten indicators because they were not clearly formulated and difficult to interpret. A further indicator was excluded due to the fact that it allowed comparisons only at regional level. As the remaining indicators did not cover all of the issues that had been defined as relevant at the beginning of the study, the indicator set was supplemented by 21 additional indicators. These particularly covered long-term care provision, participation and physical functioning. This resulted in an indicator set comprising 66 potential indicators for inclusion in the structured consensus-based process. These indicators were attributed to the predefined topics as follows: health care (9), nursing and community care (8), physical environment (3), social environment (4), social participation (4), activities of daily living (2), physical health (9), mental health (10), physical functioning (11), cognitive functioning (2) and health behaviour (4).

During the first stage of the evaluation, 25 of the 66 indicators were classified as highly relevant and 24 as relevant. The remaining 17 indicators were rated as of medium relevance and were excluded from the next stage of the evaluation. This led to 49 indicators for the second stage of evaluation.

The feedback provided during the second stage resulted in 33 indicators being classified as highly relevant, 13 indicators as relevant and 3 indicators as of medium relevance. During a workshop that preceded this stage of the evaluation, the participants had decided to make a number of modifications to the indicator set. This resulted in the combination of three potential indicators (‘recipient of inpatient care’, ‘recipient of outpatient care’ and ‘level of long-term care needs’) to form a single indicator (‘recipient of long-term care’), and the addition of a further indicator – ‘psychotropic medication’. Finally, five out of the 17 indicators classified as of medium relevance during the first stage (‘influenza vaccination’, ‘pneumococcal vaccination’, ‘pressure sores’, ‘difficulty walking’ and ‘grip strength’) were to be re-evaluated after a review of the scientific evidence.

The indicators that were not classified as highly relevant were excluded prior to the third and final stage. This process resulted in 37 indicators being selected. Information on these was documented in accordance with the schema described above (definition, data sources, type and periodicity of the data sources, reference population, options for stratification, scientific background and references) and was presented to the experts for evaluation and comment. By the end of the third stage, 18 of the 37 indicators were still classed as highly relevant, 14 were now viewed as relevant and five as of medium relevance.

The final indicator set is available on the RKI website. With 15 indicators, most of the 18 indicators selected in the final stage of the consensus-based process can be represented by data from the nationwide health surveys conducted at the Robert Koch Institute; three of the 15 indicators can be represented both by these and by surveys conducted by external data providers. Two indicators rely on data from external studies and one indicator on data from official statistics. The indicators draw on data from the following studies: the German Health Update (GEDA) [14], the German Health Interview and Examination Survey for Adults (DEGS) [15], the German Ageing Survey (DEAS) [16], the German Oral Health Study (DMS) [17], the Socio-Economic Panel (SOEP) [18], the European Quality of Life Surveys (EQLS) [19] and the European Union Statistics on Income and Living Conditions study (EU-SILC) [20].

For five of the 18 indicators data are currently available for the population aged 65 or above, but not for the population aged 80 or above. Four of these rely on data from the German Health Interview and Examination Survey for Adults (DEGS1, 2008-2011) and are, therefore, restricted to an upper age limit of 79 [15]. A fifth indicator relies on data from the German Ageing Survey (DEAS) [16] and is limited to the age of 85. However, all of the data sources used allow for the indicators to be stratified by gender and age group (albeit with the limitations mentioned above). With the exception of the ‘recipient of long-term care’ indicator, which relies on official statistics, all other indicators can also be stratified by socioeconomic status or education.

## 4. Discussion and outlook

With the final indicator set, we hope to contribute towards building a sustainable and reliable health reporting for older age in Germany. All the 18 indicators selected in the final stage can be presented on a national level using adequate and sustainable data sources. Almost all of the indicators rely on primary data collected by the Robert Koch Institute or other research institutions; just one of the indicators (‘recipient of long-term care’) can be presented using routine data. Primary data are survey or examination data that have been collected mainly for scientific purposes. Routine data include administrative data from health insurance and other social insurances as well as data from official statistics such as long-term care and cause of death statistics. The combination of primary and routine data is particularly valuable [21]. Primary data not only enable indicators to be stratified by gender or age group, but also by socioeconomic status. In addition, subjective health outcomes, such as health-related quality of life and subjective care needs, can only be displayed using survey data. On the other hand, routine data are not affected by non-response bias and they enable indicators to be updated periodically. Besides, they are not affected by age restrictions often applied to interview and examination surveys [15, 16], because older adults, especially older adults in poor health, are harder to reach by conventional recruitment and survey methods [22, 23]. Data for five of the 18 indicators selected for this set are available with an upper age limit of 79 or 85.

The integration of routine data (research data sets collated by statutory health insurers that are made available due to Germany’s Data Transparency Regulations, DaTraV) provides for better representation of indicators related to health care provision and the possibility to display the indicators on a regional level, at least down to the federal state level. The data sources currently available allow only limited regional analysis of the 18 indicators. Here, examples from other countries demonstrate that merging indicators based on data collected at the national and regional level is technically possible and creates synergies [4]. Efforts are also being made to expand federal health reporting and reporting at the federal state level in Germany along similar lines [24, 25].

The approach to select indicators using a modified Delphi technique supplemented by a full-day workshop during which questions raised by the participants could be discussed and clarified, proved to be both effective and time-saving. However, the participants found the task of using various criteria to assess indicators in accordance with a single globally valid score challenging. Future consensus processes might ask participants to assess criteria seperately, and, therefore, focus solely on the subject areas in which they have the most expertise. One limitation of the study is the fact that albeit representatives of different professions and institutions from practice and research participated in the selection process, the approach was mainly expert-led and older people’s views had limited influence during the development of the indicator set. In the future, it may be useful to base selection processes more along the lines of the approach used to draw up Ireland’s national positive ageing indicator set [5] – in addition to stakeholders from science and practice, older people participated equally as participants in the Delphi technique. Similarly, the International Consortium for Health Outcomes Measurement (ICHOM) also chose a different approach to selecting relevant health care measures: it combined an expert-led Delphi technique with a focus group of older people to ensure that their views were taken into account during the selection process [26].

No claims are made as to the comprehensiveness of the indicator set presented here, nor is it assumed that the set fully captures the health and well-being of older people. Rather, this is an ongoing process, and the indicator set will need to be adapted to account for new health and methodological challenges. Nevertheless, agreement on a conceptual framework as well as on relevant indicators for public health monitoring of the population over the age of 65 means that it is now possible to conduct comparable assessments of the health of older people over time. The aim is also for these indicators to be integrated into an overall approach to the development of health monitoring and health reporting in the prevention of chronic diseases and to ensure good health in all stages of life in accordance with international action plans [27, 28]. The development and implementation of the national diabetes surveillance, which began in 2016, has laid valuable foundations for this undertaking [29]. Finally, in the long term, a data structure needs to be established that can be used to support policy decision-making processes, the evaluation of health goals and policy impact assessments.

## Key statements

- The selection of relevant indicators is an important step in the development of continual public health monitoring of older people.
- Indicator-based public health monitoring enables comparable assessments to be made of the health of older people over time and can be used to support policy-related decision-making.
- The IMOA project selected its indicators by systematically compiling indicators from existing international monitoring systems.
- The final set of indicators on the health of older people comprises 18 indicators that are to be continuously developed further.
- The indicators are to be integrated into a future overall approach to the monitoring of chronic diseases by the Robert Koch Institute.

## Figures and Tables

**Table 1:** Health areas, domains, and indicators for public health monitoring for the 65+ age group Own diagram

Indicators	Available data sources
**Environmental factors**	
**Health care**	
Unfulfilled care needs	German Health Update (GEDA)
	European Union Statistics on Income and Living Conditions (EU-SILC)
Dental care	German Health Update (GEDA)
	German Oral Health Study (DMS)
Psychotropic medication*	German Health Interview and Examination Survey for Adults (DEGS1)
**Nursing and community care**	
Recipient of long-term care	Nursing care statistics from the Federal Statistical Office
Caregiver burden*	German Health Interview and Examination Survey for Adults (DEGS1)
**Physical environment**	
Access to important infrastructure	European Quality of Life Surveys (EQLS)
**Social environment**	
Social support	German Health Update (GEDA)
	German Health Interview and Examination Survey for Adults (DEGS1)
Loneliness**	German Ageing Survey (DEAS)
**Activities and participation**	
**Activities of daily living**	
Restrictions in activities of daily living	German Health Update (GEDA)
Restrictions in instrumental activities of daily living	German Health Update (GEDA)
**Personal factors**	
**Physical health**	
Subjective health	German Health Update (GEDA)
	German Health Interview and Examination Survey for Adults (DEGS1)
	European Union Statistics on Income and Living Conditions (EU-SILC)
	Socio-Economic Panel (SOEP)
Multimorbidity	German Health Update (GEDA)
	German Health Interview and Examination Survey for Adults (DEGS1)
**Mental health**	
Depressive symptoms	German Health Update (GEDA)
	German Health Interview and Examination Survey for Adults (DEGS1)
Life satisfaction	German Health Update (GEDA)
**Physical functioning**	
Pain	German Health Update (GEDA 2013s special survey)
Falls*	German Health Interview and Examination Survey for Adults (DEGS1)
Urinary incontinence	German Health Update (GEDA)
**Cognitive functioning**	
Cognitive impairments*	Additional mental health module of the German Health Interview and
	Examination Survey for Adults (DEGS1)

Data only available up to a specified age limit:

^*^ Available for the age group 65 to 79 years;

^**^ Available for the age group 65 to 85 years

## Appendix

**Annex Table 1:** Participants of the expert panel on indicator selection for public health monitoring of the 65+ age group

Prof Dr Michael Bosnjak	Leibniz Institute for Psychology Information Trier
Min Dir a.D. Rudolf Herweck	Federal Association of Senior Citizens Organizations, Bonn
Prof Dr Josefine Heusinger	Institute for Gerontological Research, Berlin
PD Dr Nils Lahmann	Charité – Universitätsmedizin Berlin
Prof Dr Gabriele Meyer	Martin Luther University Halle-Wittenberg
Prof Dr Ursula Müller-Werdan	Charité – Universitätsmedizin Berlin
Prof Dr Kilian Rapp	Robert Bosch Hospital, Stuttgart
Prof Dr Steffi Riedel-Heller, MPH	Leipzig University
Prof Dr Martina Schäufele	Mannheim University of Applied Sciences
Prof Dr Martin Scherer	University Hospital Hamburg-Eppendorf
Prof Dr Clemens Tesch-Römer	German Centre of Gerontology, Berlin
Prof Dr Hans-Werner Wahl	Heidelberg University
Prof Dr Karin Wolf-Ostermann	University of Bremen
Prof Dr Susanne Wurm	Friedrich Alexander University Erlangen-Nuremberg
Prof Dr Susanne Zank	University of Cologne
Dr Stephanie Heinrich (as of stage 2)	Martin Luther University Halle-Wittenberg
Dr Dagmar Lühmann (as of stage 2)	University Hospital Hamburg-Eppendorf

**Annex Table 2:** Results of the structured consensus-based process used to select indicators for public health monitoring of the 65+ age group
